# Insights on the deep carbon cycle from the electrical conductivity of carbon-bearing aqueous fluids

**DOI:** 10.1038/s41598-021-82174-8

**Published:** 2021-02-12

**Authors:** Geeth Manthilake, Mainak Mookherjee, Nobuyoshi Miyajima

**Affiliations:** 1 Laboratoire Magmas et Volcans CNRS, IRD, OPGC, Université Clermont Auvergne, 63000 Clermont-Ferrand, France; 2 Earth Materials Laboratory, Department of Earth, Ocean and Atmospheric Sciences, Florida State University, Tallahassee, FL 32306 USA; 3 Bayerisches Geoinstitut, University of Bayreuth, 95447 Bayreuth, Germany

**Keywords:** Solid Earth sciences, Geochemistry, Geophysics, Mineralogy

## Abstract

The dehydration and decarbonation in the subducting slab are intricately related and the knowledge of the physical properties of the resulting C–H–O fluid is crucial to interpret the petrological, geochemical, and geophysical processes associated with subduction zones. In this study, we investigate the C–H–O fluid released during the progressive devolatilization of carbonate-bearing serpentine-polymorph chrysotile, with in situ electrical conductivity measurements at high pressures and temperatures. The C–H–O fluid produced by carbonated chrysotile exhibits high electrical conductivity compared to carbon-free aqueous fluids and can be an excellent indicator of the migration of carbon in subduction zones. The crystallization of diamond and graphite indicates that the oxidized C–H–O fluids are responsible for the recycling of carbon in the wedge mantle. The carbonate and chrysotile bearing assemblages stabilize dolomite during the devolatilization process. This unique dolomite forming mechanism in chrysotile in subduction slabs may facilitate the transport of carbon into the deep mantle.

## Introduction

Subduction is one of the key processes that regulate the transport of water and carbon into the Earth’s mantle^1–3^. It is estimated that the extreme slab dehydration may release 97% of the water bound in hydrous minerals into the overlying mantle^4^, while 80% of the carbonates in subducting slabs may decarbonate and be recycled through arc volcanism^5,6^. The flux of volatiles released by the slab brings about dramatic changes to the physical and chemical state of the overlying mantle, including the melting temperatures^7^, redox state^8,9^, and mechanical strength of mantle minerals^10,11^. The upward migration of volatiles associated with dehydration and decarbonation is therefore key to understand the petrological, geochemical, and geophysical processes associated with subduction zones^12^.

The investigations of slab-devolatilization reveal close affinity between the transfer of slab bound carbon to the mantle wedge and the dehydrating aqueous fluids^13–16^. The dissolution of carbon in slab-derived aqueous fluids displays a complex dependence of pressure, temperature, and redox conditions^8,17^. It has been shown that carbonate solubility in water increase with pressure, implying that at deep mantle conditions, carbonates may readily dissolve in aqueous fluids^17^. The carbon dissolved in aqueous fluids in equilibrium with mantle peridotites mainly occurs as molecular CO_2_^14^, however, depending on the prevailing conditions, carbon can be dissolved in aqueous fluids as methane (CH_4_)^13,16,18^. A recent experimental study demonstrated an immiscibility of C–H–O fluids above 1.5 GPa and ~ 900 K, which may result in immiscible C–H–O fluids in the mantle wedge^13,18,19^.

In addition to the petrological and geochemical studies which provide constraints on the chemical interactions of fluids with the surrounding mantle, geophysical methods such as magnetotelluric and seismic tomography also provide complementary constraints on the volume fraction of the fluid, and mobility of the fluid in the mantle wedge^20–24^.

The laboratory-based constraints on the electrical conductivity of fluids have provided valuable insight into the fluid-driven processes in the subduction zone and overlying mantle wedge^23, 24^. The experimental constraints on the electrical conductivity of aqueous fluid include pure water^25,26^, aqueous fluids released by dehydration^23,24,27,28^ and saline aqueous fluids with varying salinities^29–34^. However, the effect of both carbon and hydrogen bearing (C–H–O) aqueous fluids on electrical conductivity remain unknown.

**Figure 1:**
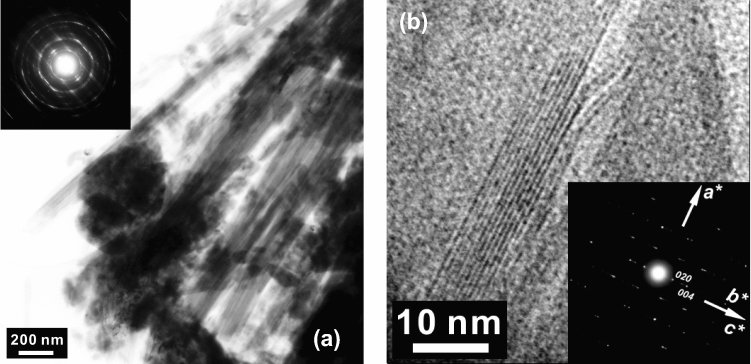
The characterization of the chrysotile sample. (**a**) Bright-field TEM image of fibrous chrysotile. The inset is a selected area electron diffraction (SAED) pattern from the fibrous crystals. (**b**) The high-resolution TEM image of the fibrous chrysotile showing lattice fringes of about 0.7 nm. The inset is the corresponding SAED pattern of a mixture of the [010] and [001] zone axes due to the cylindrical structure.

Hence, to bridge this crucial gap, in this study, we constrain the electrical conductivity of C–H–O fluids. To measure the electrical conductivity of C–H–O fluids we chose a naturally occurring carbonate-bearing serpentinite which consists of chrysotile (Fig. 1). Chrysotile is a polymorph of serpentine. Owing to its importance in subduction zone settings, extensive studies on the equation of state^35^, elasticity^36–39^, and electrical conductivity^28,40,41^ have been conducted on serpentine polymorphs including lizardite and antigorite. Relative thermodynamic stability of chrysotile is lizardite suggest that chrysotile less stable compared to lizardite, however, in nature, chrysotile and lizardite do occur at similar pressure and temperature conditions, often at very low-grade metamorphic conditions relevant for oceanic serpentinization^42^. The chrysotile found in nature is metastable and it is often favored in tectonically active regions^42^. Although chrysotile is metastable, since the focus of this study was to understand the electrical conductivity of IC–H–O fluids, we chose a carbonate-bearing chrysotile sample for the temperature-dependent electrical conductivity measurements. The temperature was incrementally increased beyond the thermodynamic stability field of the hydrous and carbonate phases leading to the release of aqueous fluids that would mimic C–H–O fluids occurring in the subducting slab^13^. To evaluate the effect of carbon on the electrical conductivity of the aqueous fluid, we conduct a separate experiment where we measure another  serpentine (antigorite) without any detectable presence of carbonate phases. The aqueous fluids released upon dehydration of antigorite serves as a control experiment without the effect of carbon.

## Results

**Figure 2:**
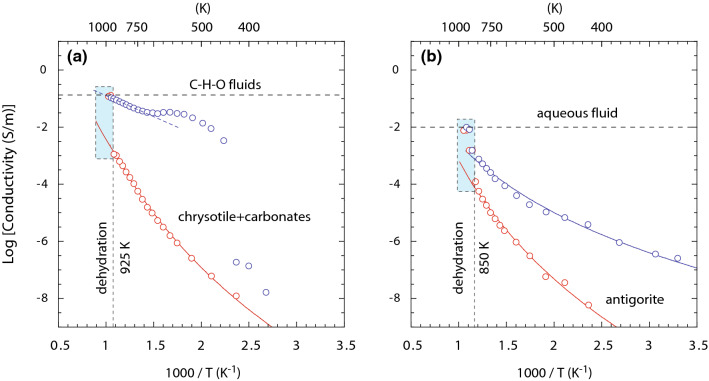
The electrical conductivity of serpentine as a function of reciprocal temperature for (**a**) chrysotile and (**b**) antigorite. The red open circles represent the electrical conductivity of the serpentine samples collected during the heating path (i.e. with increasing temperature). The blue open circles indicate electrical conductivity obtained in the cooling path. The dashed vertical lines indicate the observed devolatilization temperatures determined based on the discontinuous enhancements in the electrical conductivity. The blue shaded area indicates the conductivity increase associated with the devolatilization. The horizontal lines indicate the maximum electrical conductivity observed for released aqueous fluids upon dehydration of the two samples.

Upon heating serpentine samples from 300 K to the dehydration temperatures ~ 850–925 K, the electrical conductivity increases from 10^–8^ to 10^–4^–10^–3^ S/m respectively (Fig. 2). The dehydration of both serpentinite samples i.e., with and without carbonate phase, resulted in a discontinuous increase of conductivity. The aqueous fluid resealed from both the serpentinite samples exhibits more than two orders of magnitude higher electrical conductivity compared to the electrical conductivity prior to the dehydration. A sharp increase in electrical conductivity occurs at 850 K for antigorite polymorph that also contains magnetite. Such a sharp increase in electrical conductivity occurs at ~ 925 K for chrysotile polymorph containing calcite. The temperature dependence of the electrical conductivity is well described by an Arrhenius relation; $\sigma ={\sigma }_{0}\mathrm{exp}(-\Delta \mathrm{H}/RT)$. And the logarithmic dependence of electrical conductivity $\mathrm{ln}(\sigma$) versus the reciprocal temperature ($1/T$) yields the activation enthalpy ($\Delta \mathrm{H}$) of the dominant conduction mechanisms. The conductivity ($\sigma$) is expressed in S/m, temperature ($T$) is in absolute temperature, ${\sigma }_{0}$ is the pre-exponential factor in S/m, and $R$ is the gas constant in J K^−1^ mol^−1^. We obtain the activation enthalpy and the pre-exponential factor following the combined electrical conductivity ${\sigma }_{total}={\sigma }_{m1}+{\sigma }_{m2}$^43,44^. The notations ${m}_{i}, i=\mathrm{1,2}$, indicates distinct conduction mechanisms operating at different temperature intervals^45^ (Table 1). At low temperatures, < 600 K, the electrical conduction is likely to be dominated by proton conduction. One possibility could be the presence of protons in the grain boundaries that were not fully removed when the sample was kept overnight for removing the absorbed moisture. Similar observations have been made in the electrical conductivity of deformed hydrous silicates including talc and serpentinites^45^. At the intermediate temperature range i.e., between 600 and 900 K, the electrical conduction shows slightly higher activation enthalpy. This suggests a possible contribution from polarons as the charge carrier^45^. In contrast, after dehydration the electrical conductivity shows weak temperature dependence, indicating the involvement of the free fluid for the conduction^23,24^.

**Table 1:** Fitting parameters for electrical conductivity of serpentine and dehydrating fluids.

Sample	Temperature (K)	Log σ_0_ (S/m)	ΔH (eV)
Chrysotile	< 600	− 1.7	0.75
	600–925	1.74	0.82
Chrysotile + C–H–O Fluid	975–720	0.57	0.21
Antigorite	< 623	− 2.7	0.73
	623–850	3.76	0.83
Antigorite + Aq-fluid	825–673	2.4	0.8
	673–300	− 2.3	0.7

**Figure 3:**
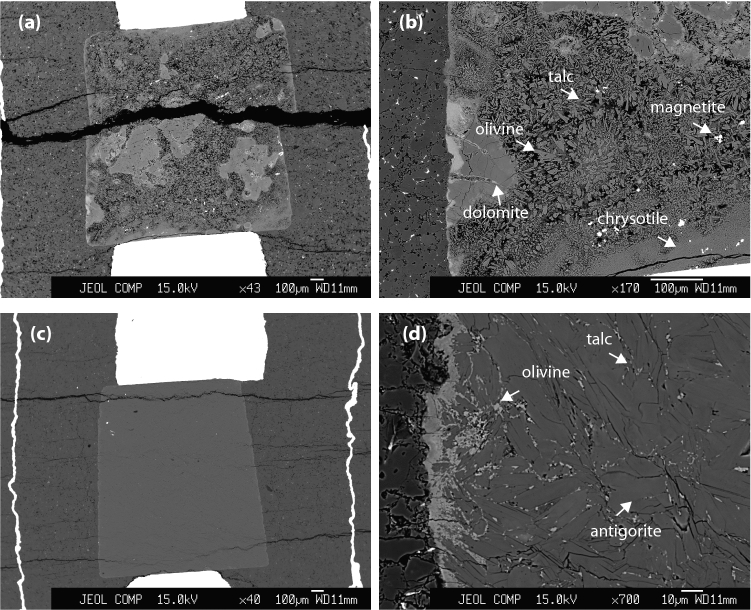
Back Scattered Electron (BSE) images showing the cross-section of the (**a**,**b**) chrysotile and (**c**,**d**) antigorite samples after electrical conductivity measurements, cut along the axial direction of the assembly.

**Figure 4:**
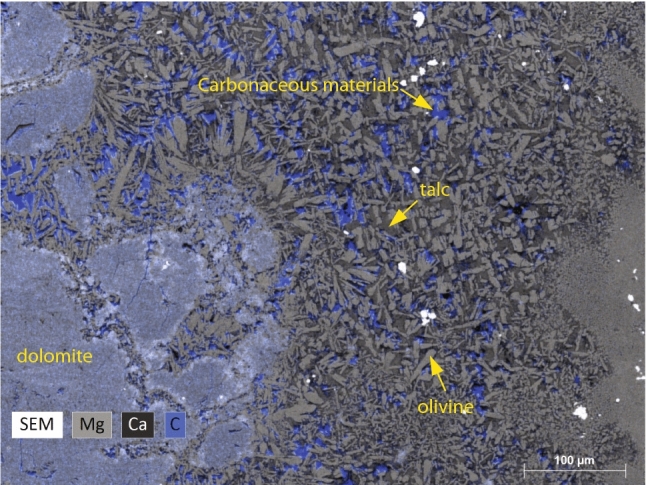
Back Scattered Electron (BSE) image at higher magnification, shows the dehydrated carbonate-bearing chrysotile sample. The BSE image is overlain with the energy-dispersive X-ray (EDX) spectroscopy which shows the mapping of Mg (light gray), Ca (dark gray), and C (blue). The concentrations of blue regions indicate precipitation of carbonaceous materials within the olivine and talc grain boundaries.

The chemical analysis and BSE images of samples after electrical conductivity measurements indicate that the serpentine mineral decomposes to a mixture of olivine and talc, and the released aqueous fluid is evident from the voids (Fig. 3). The decomposition of the chrysotile sample and exhibits extensive dolomite crystallization possibly triggered by the exchange of Ca^2+^ and Mg^2+^ between calcite and serpentine (Fig. 3). In addition, the chrysotile sample displays precipitation of carbonaceous matter along the boundaries of olivine and talc grains (Fig. 4). At similar pressure and temperature conditions, the antigorite sample did not show dolomite crystallization, since it did not have any carbonate phase prior to dehydration.

## Discussion

### Chemical characterizations of the fluid phase

The in-situ electrical conductivity measurements cannot distinguish individual contributions from the chemical species of C–H–O fluid as it represents the bulk conductivity of the interconnected fluid phase. Here we probe the nature of the fluid produced by the devolatilization of carbonate baring chrysotile by both Energy dispersive X-ray diffraction (EDX) chemical mapping and micro Raman spectral analyses of experimental run product.

**Figure 5:**
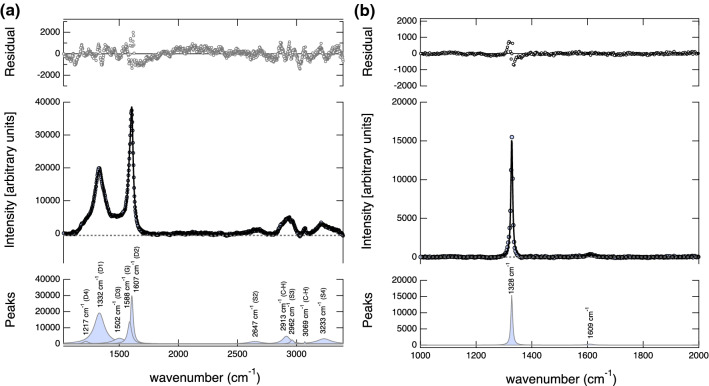
Raman spectra of carbonaceous materials after the devolatilization of the carbonate-bearing chrysotile sample. (**a**) Deconvolution of Raman spectra shows graphite and C–H stretching modes likely indicating CH_4_. The vibrational modes are characterized by first-order bands of ordered graphite at 1588 cm^−1^ (G) and disordered graphite bands at 1217 cm^−1^ (D4) , 1332 cm^−1^ (D1), 1502 cm^−1^ (D3) and 1607 cm^−1^ (D2)^46^. The second-order overtones of graphite bands are less intense compared to the primary modes and are observed at 2647 cm^−1^ (S2), 2962 cm^−1^ (S3), and 3233 cm^−1^ (S4)^46^. Additional bands are also identified at 2913 cm^−1^ and 3069 cm^−1^ and are likely due to C–H stretching^50^. (**b**) Raman spectrum showing an intense and sharp mode at ~ 1328 cm^−1^ indicative of crystalline diamond^48^ which may have formed in the C–H–O fluid.

The micro-Raman analyses of the chrysotile sample after dehydration reveal characteristic vibrational modes corresponding to graphite^46,47^, diamond^15,48^, and methane (CH_4_)^49–51^ (Fig. 5). The precipitation of carbonaceous matter along the grain boundaries of olivine and talc, formed as a result of the dehydration of chrysotile, suggests that the resulting fluid phase was likely rich in CO_2_. The precipitation of carbonaceous materials such as graphite and diamond suggests following the reaction: CO_2_ = C + O_2,_ and CO_2_ + 2H_2_O = CH_4_ + 2O_2_. The liberation of O_2_ indicates the oxidizing nature of the released fluid. Although the starting composition of the chrysotile and carbonates were devoid of magnetite (Fe_2_O_3_), the oxidizing nature of the released aqueous fluids leads to the formation of Fe_2_O_3_ in the chrysotile sample after the dehydration (Fig. 4). The formation of Fe_2_O_3_ occurs concomitantly with the precipitation of C or CH_4_ by the decomposition of CO_2_ by the above-mentioned reactions. The formation of Fe^3+^ bearing Fe_2_O_3_ and the Raman modes on the carbonaceous precipitates are clearly indicative of the oxidizing nature of the released fluids. The oxidizing properties of the slab-derived fluids conforms with the highly oxidized nature observed in arc magmas compared to mid-ocean ridge basalts^52,53^.

The presence of diamonds in the experimental product was confirmed by Raman analyses. Prior to the Raman analyses, the spectral position was calibrated using synthetic low-fluorescence diamond oriented in the 100 direction (Boehler-Almax). The presence of a diamond peak at 1328 (± 0.02) cm^−1^ agrees well with the vibrational modes ~ 1332–1328 observed for diamonds crystallized from C–H–O fluids^48^. The precipitation of diamond at temperatures < 1200 K, suggests either CH_4_ = C + 2H_2_ or CH_4_ + O_2_ = C + 2H_2_O^51^ or saturation of carbon in the fluid phase^48^. Our study provides direct experimental evidence relating to the origin of subduction-related micro diamonds found in ultrahigh-pressure metamorphic terrains^49,54^.

### Wetting properties of the fluid phase

In a multiphase system, the electrical conductivity is governed by the highest conductive phase that can develop an interconnected network within the matrix. In the case of aqueous fluids, the development of the interconnected network at low volume fractions depends on their wetting properties which are manifested as the dihedral angle of the fluids in the interstitial pockets formed within the framework of grains. If the dihedral angle, θ < 60°, the fluid is likely to form an interconnected network and percolate through the mineral grain boundaries even at small volume fractions. In contrast, if the θ > 60°, the fluid is likely to be trapped in the interstitial pockets and percolation is only possible for high fluid fractions. At pressures < 4 GP and temperatures < 1073 K, prior experimental investigations of aqueous fluids have shown limited wetting properties i.e., θ > 60°^55–58^, which is likely to restrict the connectivity of aqueous fluids. In comparison to aqueous fluids, CO_2_-rich fluids exhibit larger dihedral angles with 80 < θ < 120°^59,60^, thus severely restricting their movement along grain boundaries. The presence of C–H–O fluid, therefore, should not necessarily result in a high electrical conductivity, unless the fluid fraction is large to overcome the limited wetting property and establish a network of interconnected fluid. The dihedral angle of fluid-filled grain boundaries was measured after the dehydration of chrysotile and antigorite and it exhibits distinct variations, which may affect their ability to percolate into a polycrystalline matrix. The observed θ for the olivine-fluid-olivine interfaces produced by carbonate-bearing chrysotile vary between 45° and 60°, in contrast, observed θ produced by the dehydrated antigorite is 0–10°. The contrast in wetting properties observed in these two experiments are likely related to the distinct nature of the C–H–O fluids and carbon-free aqueous fluids^61^. This is the likely cause of the notable differences observed in the temperature dependence of the electrical conductivity (Fig. 2). In the antigorite sample, the conductivity increase due to dehydration occurs at 850 K, which can be explained by the development of interconnected fluid network at low fluid fractions due to their excellent wetting properties. With the gradual increase of temperature, electrical conductivity increases in response to the increase of fluid volume fraction in the antigorite sample. In contrast, in the chrysotile sample, the discontinuous increase of conductivity occurs at 925 K. The conductivity enhancement at higher temperatures is likely due to the non-wetting of grain boundaries at a low fluid fraction. Once the fluid fraction is large enough in the chrysotile sample, the network of interconnected fluids is established, resulting in a sharp increase of conductivity of more than two orders of magnitude. The conductivity behavior in the cooling path often indicates the nature of the dominant conductive phase. The relatively high electrical conductivity of the and weaker temperature dependence observed in carbonate-bearing chrysotile sample is likely caused by the precipitation of the conductive graphite phase along the grain boundaries. Similar behavior of relatively high electrical conductivity and weak temperature dependence in aqueous fluids have been previously observed in dehydration of chlorite where conductive magnetite phases were precipitated along grain boundaries^24^.

### Highly conductive C–H–O fluid tracks deep carbon

**Figure 6:**
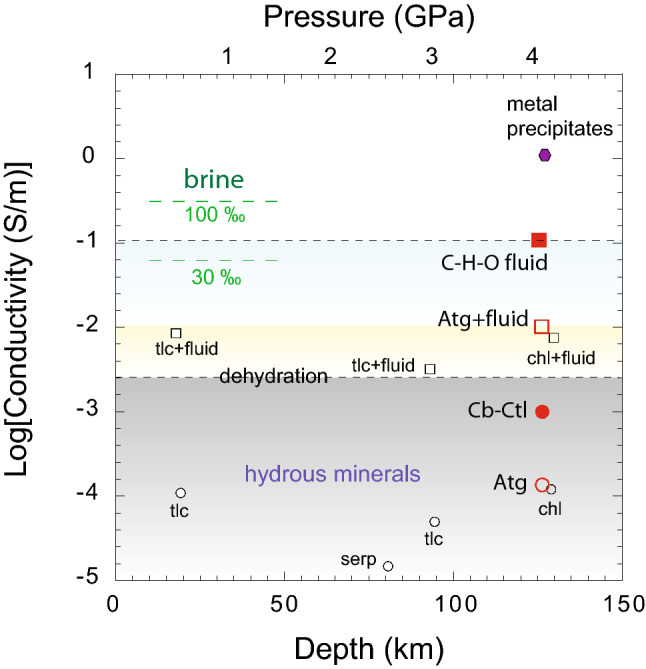
A compilation of electrical conductivity data of layered hydrous silicate minerals and released aqueous fluids upon dehydration and metal precipitates that are often facilitated by dehydration. The electrical conductivity data reported in this figure are from previously reported electrical conductivity studies on talc (tlc)^45,83^, serpentine (serp), and chlorite (chl)^24^. The electrical conductivity of hydrous minerals, aqueous fluids released via dehydration in subduction-zone settings, and metal precipitates are segregated by dashed horizontal lines. It also shows that the electrical conductivity of C–H–O aqueous fluids is quite high compared to the normal aqueous fluids. However, the formation of interconnected networks of metal precipitates is likely to generate the highest electrical conductivities in subduction zone settings^24^.

The temperature dependence of the electrical conductivity of serpentine agrees well with prior studies on natural serpentinite rock^45^, the polymorph of serpentine-antigorite^41,62^, and lizardite^28^. The fluids released during the dehydration of serpentine exhibit ~ 2 orders of magnitude higher conductivity than their host minerals phases. The high conductivity of aqueous fluids compared to dehydrated mineral residue has been observed in other studies^23,24^. The high conductivity can be explained by the increased mobility of charge carriers in the aqueous fluid medium. The electrical conductivity of C–H–O fluids displays more than one order of magnitude greater electrical conductivity compared to C-free aqueous fluids. The elevated conductivity of C–H–O fluids can be attributed to the high concentrations of dissolved mobile ions in the resulting fluid. The contrast in electrical conductivity may provide diagnostic criteria for detecting the dehydrating C–H–O fluids that carry carbon into the mantle wedge (Fig. 6).

### Implications for the carbon recycling of the mantle

The low carbon concentrations in the mantle compared to the solar abundances^63,64^ may indicate either owing to its volatile nature, carbon was lost during accretion stages in the early history of the Earth or it is also likely that a part of this missing carbon might be sequestered in the Earth’s interior^5^. However, unlike hydrogen, which is often sequestered as defects in nominally anhydrous mineral phases^65^, the solubility of carbon in mantle silicate is extremely limited^66,67^, and hence, in the deep mantle carbon mainly occurs as accessary carbonate minerals stabilized in deeply subducted slabs^66,68^. It has been suggested that high-pressure dolomite (dolomite-III)^69^ and magnesite are the major carbonate phase that is stable in the Earth’s lower mantle^69–73^. The stability of magnesite depends on the mantle redox conditions, at highly reducing conditions, magnesite is likely to decompose and transform into a mixture of diamond or metal carbides^74^. However, the transport of carbonates into the mantle transition zone and the lower mantle could still occur through deeply subducted slabs, where relatively oxidized environments are likely^74^.

Based on the seismic velocity, it is expected that 20–45% of the ocean floor is serpentinized by hydrothermal alterations^75^. As the volumetrically important hydrated lithology in the subducting slab, serpentine plays a vital role in transporting water into the deep mantle, however, its significance as a host for carbonate/carbon and the role it is likely to play in carbon sequestration in the deep Earth is equally crucial. The CO_2_-rich fluids released during the devolatilization of carbonate-bearing chrysotile present ideal conditions to stabilize dolomite or magnesite^76^, thus providing an efficient mechanism to transport carbon to the deep mantle via deeply subducted altered oceanic crust.

In our experimental study, we observe extensive crystallization of dolomite following the dehydration of calcite bearing chrysotile. It has been shown that dolomite can be transformed into magnesite bearing phases at high pressure^70^ or can be transformed into high-pressure phases such as dolomite-III above 35 GPa^69^. The dolomite forming reaction during the dehydration decarbonation of carbonate-bearing serpentine is therefore a key process in transporting carbon into the deep Earth by subducted slabs.

## Methods

### Sample characterization

In this study, we used two serpentinite samples. One of the serpentinite contained chrysotile polymorph and minor amounts of calcite. The other serpentinite sample consists dominantly of antigorite polymorph with a minor amount of magnetite (Supplementary Fig. 1). The chemical composition of each sample, both before and after the electrical conductivity measurements were confirmed with an electron probe microanalysis (EPMA) using a JEOL-JXA-8200 electron microprobe operating at an accelerating voltage of 15 kV and a beam current of 20 nA (Supplementary Table 1). The chemical mapping of the samples was obtained using energy dispersive x-ray spectroscopy (EDS) chemical mapping using a JEOL JSM-5910LV scanning electron microscope (SEM) The chrysotile starting sample was further characterized by a transmission electron microscope (TEM) equipped with a field emission gun operated at 200 kV at Bayerisches Geoinstitut, Germany (Fig. 1). TEM often provides details about the microstructure, texture, and structural relationship in serpentine polymorphs^77^. Also, the crystal structure determinations of chrysotile is often challenging and rely on modeling^78^, TEM has often been used to identify chrysotile. For chemical analyses, samples were polished along the axial direction of the high-pressure cell using corundum powder. During the sample preparation for EMPA and SEM analyses, we avoid the traditional carbon coating of the sample, instead, coated the sample with a gold layer to counter the charging effect. These strict protocols help avoid the undesired contaminations of carbon-based compounds during the sample preparation.

High-resolution SEM images of the plane of the polished cross-section of the sample^79,80^ were used to determine the grain boundary/wetting angles. The apparent angles were measured by placing two vectors along the two adjacent grain boundaries that were in contact with the fluid. For each sample analyzed, we have measured ~ 100 apparent angles. The precision of the angle measurements is within ± 5°.

### High-pressure and temperature experiments

High-pressure, high-temperature experiments were performed using a 1200-ton multi-anvil apparatus at Bayerisches Geoinstitut, Germany. We use a 25/15 multi-anvil cell configuration for the electrical conductivity measurements at 4 GPa (Supplementary Fig. 1). For the electrical conductivity measurements, cylindrical cores of serpentine samples with 2 mm diameter and 2.5 mm in length were placed in a polycrystalline MgO capsule, which electrically insulates the sample from the furnace during the measurements. Two molybdenum (Mo) disks placed at the top and bottom sides of the sample served as electrodes for the electrical conductivity measurements. The presence of Mo adjacent to the sample is also expected to maintain the oxygen fugacity of the sample close to the Mo–MoO_2_ buffer. A Mo metal-foil jacket (25 μm) placed between the graphite furnace and the sample protect the sample from possible contaminations from graphite diffusing through the MgO-sleeve at high temperature. The metal jacket, connected to the ground via an anvil also removes undesirable electrical noise generated by the heating system, improving the quality of impedance spectra. A W_95_Re_5_–W_74_Re_26_ thermocouple junction was placed at one side of the sample, which monitored the temperature. One cable from the thermocouple and a separate W_95_Re_5_ cable connected to the opposite side of the sample connected the impedance spectroscope for the electrical conductivity measurements. The three-electrode configuration avoids lead cables sharing the same anvil, which may improve the insulation resistance of the assembly. MgO ceramic sleeves insulate the electrode wires and avoid undesired contacts with the furnace. All ceramic assembly parts including the pressure media were baked at 1273 K for more than 12 h to remove adsorbed moisture from the ceramic assembly components.

### Impedance spectroscopy

Electrical conductivity measurements were based on the impedance spectroscopy method using the Solatron 1260 Impedance/Gain-phase analyzer in the frequency range of 10^6^–10^1^ Hz. We have determined the insulation resistance of the assembly at similar pressure–temperature conditions prior to the actual experiment to determine the lowest resistance that can be measured using the present assembly configuration. After reaching the desired pressure, we kept the assembly at 500 K for more than 12 h to remove adsorbed moisture from the surrounding assembly parts. The next heating cycle was started once the sample resistance reached a steady value^81^.

The measurements were conducted in several heating–cooling cycles. Once the electrical conductivity of the starting samples was established, the temperature gradually increased to initiate the dehydration of the sample, and measurements were performed in smaller temperature steps (25–50 K). Once the interconnectivity of the fluid phase has been completed, as seen by the drastic decrease of the sample resistance, the temperature gradually brought down to room temperature in similar temperature steps, while collecting the impedance spectra at each step.

The impedance spectra of polycrystalline samples can be characterized by a combination of resistor–capacitor (R–C/CPE) circuits. The sample resistance can be obtained by fitting impedance spectra to an appropriate equivalent circuit. As for the dry sample, resistance is dominated by grain interior processes; the fitting requires resistor–capacitor circuits in series^23,24^. However, when dehydration introduces highly conductivity fluids, the influence of the grain boundary (fluid phase) process dominates over the grain interior, so that the resister-capacitor circuits have to be in parallel configuration^23,24^. Once the sample resistance has been determined, electrical conductivity can be determined using the sample diameter and length.

### Micro-Raman analyses

Raman spectroscopy is one of the few analytical techniques that can identify structural changes in carbon. This technique is also suitable to identify solute species in fluids^50^. Raman spectra were collected in a back-scattered geometry using an InVia confocal Raman micro-spectrometer, equipped with a 532 nm diode laser, a Peltier-cooled CCD detector, a Rayleigh rejection edge filter^82^. The laser power of 1 mW; the slit aperture of 20 μm, and a grating of 12,400 l/mm were used for the present analyses. These conditions result in lateral and axial spatial resolutions of ~ 1 and 3 μm and a spectral resolution of less than 1 cm^−1^. The acquisition time was 15 s.

## Supplementary Information

Supplementary Information.

## Data Availability

All data generated or analyzed during this study are included in this article and its Supplementary Information files. The raw electrical conductivity data are available from the corresponding author on reasonable request.

## References

[1] Wallace PJ (2005). Volatiles in subduction zone magmas: Concentrations and fluxes based on melt inclusion and volcanic gas data. J. Volcanol. Geotherm. Res..

[2] Rüpke LH, Morgan JP, Hort M, Connolly JADD (2004). Serpentine and the subduction zone water cycle. Earth Planet. Sci. Lett..

[3] Rüpke LH, Morgan JP, Dixon JE, Jacobsen SD, Van Der Lee S (2006). Implications of subduction rehydration for Earth’s deep water cycle. Earth’s Deep Water Cycle.

[4] Dixon JE, Leist L, Langmuir C, Schilling J (2002). Recycled dehydrated lithosphere observed in plume-influenced mid-ocean-ridge basalt. Nature.

[5] Dasgupta R, Hirschmann MM (2010). The deep carbon cycle and melting in Earth’s interior. Earth Planet. Sci. Lett..

[6] Kelemen PB, Manning CE (2015). Reevaluating carbon fluxes in subduction zones, what goes down, mostly comes up. Proc. Natl. Acad. Sci. USA.

[7] Hirschmann MM (2000). Mantle solidus: Experimental constraints and the effects of peridotite composition. Geochem. Geophys. Geosyst..

[8] Debret B, Sverjensky DA (2017). Highly oxidising fluids generated during serpentinite breakdown in subduction zones. Sci. Rep..

[9] Piccoli F (2019). Subducting serpentinites release reduced, not oxidized, aqueous fluids. Sci. Rep..

[10] Arcay D, Tric E, Doin MP (2005). Numerical simulations of subduction zones. Effect of slab dehydration on the mantle wedge dynamics. Phys. Earth Planet. Inter..

[11] Hacker BR, Peacock SM, Abers GA, Holloway SD (2003). Subduction factory 2. Are intermediate-depth earthquakes in subducting slabs linked to metamorphic dehydration reactions?. J. Geophys. Res..

[12] Schmidt MW, Poli S (1998). Experimentally based water budgets for dehydrating slabs and consequences for arc magma generation. Earth Planet. Sci. Lett..

[13] Li Y (2017). Immiscible C–H–O fluids formed at subduction zone conditions. Geochem. Perspect. Lett..

[14] Caciagli NC, Manning CE (2003). The solubility of calcite in water at 6–16 kbar and 500–800 °C. Contrib. Mineral. Petrol..

[15] Frezzotti ML, Selverstone J, Sharp ZD, Compagnoni R (2011). Carbonate dissolution during subduction revealed by diamond-bearing rocks from the Alps. Nat. Geosci..

[16] Sverjensky DA, Stagno V, Huang F (2014). Important role for organic carbon in subduction-zone fluids in the deep carbon cycle. Nat. Geosci..

[17] Poli S, Franzolin E, Fumagalli P, Crottini A (2009). The transport of carbon and hydrogen in subducted oceanic crust: An experimental study to 5 GPa. Earth Planet. Sci. Lett..

[18] Huang F, Daniel I, Cardon H, Montagnac G, Sverjensky DA (2017). Immiscible hydrocarbon fluids in the deep carbon cycle. Nat. Commun..

[19] Kawamoto T (2013). Mantle wedge infiltrated with saline fluids from dehydration and decarbonation of subducting slab. Proc. Natl. Acad. Sci. USA.

[20] McGary RS, Evans RL, Wannamaker PE, Elsenbeck J, Rondenay S (2014). Pathway from subducting slab to surface for melt and fluids beneath Mount Rainier. Nature.

[21] Evans RL, Wannamaker PE, McGary RS, Elsenbeck J (2014). Electrical structure of the central Cascadia subduction zone: The EMSLAB Lincoln Line revisited. Earth Planet. Sci. Lett..

[22] Zhao D (2001). Seismological structure of subduction zones and its implications for arc magmatism and dynamics. Phys. Earth Planet. Inter..

[23] Manthilake G, Mookherjee M, Bolfan-Casanova N, Andrault D (2015). Electrical conductivity of lawsonite and dehydrating fluids at high pressures and temperatures. Geophys. Res. Lett..

[24] Manthilake G, Bolfan-Casanova N, Novella D, Mookherjee M, Andrault D (2016). Dehydration of chlorite explains anomalously high electrical conductivity in the mantle wedges. Sci. Adv..

[25] Hamann SD, Linton M (1966). Electrical conductivity of water in shock compression. Trans. Faraday Soc..

[26] Hamann SD, Linton M (1969). Electrical conductivities of aqueous solutions of KCl, KOH and HCl, and the ionization of water at high shock pressures. Trans. Faraday Soc..

[27] Wang D, Guo Y, Yu Y, Karato SI (2012). Electrical conductivity of amphibole-bearing rocks: Influence of dehydration. Contrib. Mineral. Petrol..

[28] Zhu M, Xie H, Guo J, Bai W, Xu Z (2001). Impedance spectroscopy analysis on electrical properties of serpentine at high pressure and high temperature. Sci. China Ser. D Earth Sci..

[29] Guo H, Keppler H (2019). Electrical conductivity of NaCl-Bearing aqueous fluids to 900 °C and 5 GPa. J. Geophys. Res. Solid Earth.

[30] Guo X, Yoshino T, Shimojuku A (2015). Electrical conductivity of albite–(quartz)–water and albite–water–NaCl systems and its implication to the high conductivity anomalies in the continental crust. Earth Planet. Sci. Lett..

[31] Shimojuku A, Yoshino T, Yamazaki D (2014). Electrical conductivity of brine-bearing quartzite at 1 GPa: Implications for fluid content and salinity of the crust. Earth Planets Space.

[32] Shimojuku A, Yoshino T, Yamazaki D, Okudaira T (2012). Electrical conductivity of fluid-bearing quartzite under lower crustal conditions. Phys. Earth Planet. Inter..

[33] Sinmyo R, Keppler H (2017). Electrical conductivity of NaCl-bearing aqueous fluids to 600 °C and 1 GPa. Contrib. to Mineral. Petrol..

[34] Sun W (2020). Electrical conductivity of clinopyroxene–NaCl–H_2_O system at high temperatures and pressures: Implications for high-conductivity anomalies in the deep crust and subduction zone. J. Geophys. Res. Solid Earth.

[35] Hilairet N, Daniel I, Reynard B (2006). P–V equations of state and the relative stabilities of serpentine varieties. Phys. Chem. Miner..

[36] Bezacier L, Reynard B, Bass JD, Wang J, Mainprice D (2010). Elasticity of glaucophane, seismic velocities and anisotropy of the subducted oceanic crust. Tectonophysics.

[37] Marquardt H, Speziale S, Koch-Müller M, Marquardt K, Capitani GC (2015). Structural insights and elasticity of single-crystal antigorite from high-pressure Raman and Brillouin spectroscopy measured in the (010) plane. Am. Mineral..

[38] Mookherjee M, Stixrude L (2009). Structure and elasticity of serpentine at high-pressure. Earth Planet. Sci. Lett..

[39] Mookherjee M, Capitani GC (2011). Trench parallel anisotropy and large delay times: Elasticity and anisotropy of antigorite at high pressures. Geophys. Res. Lett..

[40] Stesky RM, Brace WF (1973). Electrical conductivity of serpentinized rocks to 6 kilobars. J. Geophys. Res..

[41] Reynard B, Mibe K, de Moortèle BV (2011). Electrical conductivity of the serpentinised mantle and fluid flow in subduction zones. Earth Planet. Sci. Lett..

[42] Evans BW (2004). The serpentinite multisystem revisited: Chrysotile is metastable. Int. Geol. Rev..

[43] Fei H, Druzhbin D, Katsura T (2020). The Effect Of Water On Ionic Conductivity In Olivine. J. Geophys. Res. Solid Earth.

[44] Manthilake G (2020). The electrical conductivity of liebermannite: Implications for water transport into the Earth’s lower mantle. J. Geophys. Res. Solid Earth.

[45] Guo X, Yoshino T, Katayama I (2011). Electrical conductivity anisotropy of deformed talc rocks and serpentinites at 3GPa. Phys. Earth Planet. Inter..

[46] Henry DG, Jarvis I, Gillmore G, Stephenson M (2019). Raman spectroscopy as a tool to determine the thermal maturity of organic matter: Application to sedimentary, metamorphic and structural geology. Earth-Sci. Rev..

[47] Lahfid A (2010). Evolution of the Raman spectrum of carbonaceous material in low-grade metasediments of the Glarus Alps (Switzerland). Terra Nov..

[48] Frezzotti ML, Huizenga JM, Compagnoni R, Selverstone J (2014). Diamond formation by carbon saturation in C–O–H fluids during cold subduction of oceanic lithosphere. Geochim. Cosmochim. Acta.

[49] Vitale Brovarone A (2017). Massive production of abiotic methane during subduction evidenced in metamorphosed ophicarbonates from the Italian Alps. Nat. Commun..

[50] Frezzotti ML, Tecce F, Casagli A (2012). Raman spectroscopy for fluid inclusion analysis. J. Geochem. Explor..

[51] Luth RW, Stachel T (2014). The buffering capacity of lithospheric mantle: Implications for diamond formation. Contrib. Mineral. Petrol..

[52] Carmichael ISE (1991). Mineralogy and the redox states of basic and silicic magmas: A reflection of their source regions?. Contrib. Mineral. Petrol..

[53] Kelley KA, Cottrell E (2009). Water and the oxidation state of subduction zone magmas. Science.

[54] Cartigny P (2001). The origin and formation of metamorphic microdiamonds from the Kokchetav massif, Kazakhstan: A nitrogen and carbon isotopic study. Chem. Geol..

[55] Bruce Watson E, Brenan JM (1987). Fluids in the lithosphere, 1. Experimentally-determined wetting characteristics of CO_2_H_2_O fluids and their implications for fluid transport, host-rock physical properties, and fluid inclusion formation. Earth Planet. Sci. Lett..

[56] Watson EB, Brenan JM, Baker DR (1991). Continental Mantle.

[57] Mibe K, Fujii T, Yasuda A (1998). Connectivity of aqueous fluid in the Earth’s upper mantle. Geophys. Res. Lett..

[58] Holness MB (2006). Melt-solid dihedral angles of common minerals in natural rocks. J. Petrol..

[59] Holness MB, Graham CM (1991). Equilibrium dihedral angles in the system H_2_O–CO_2_–NaCl–calcite, and implications for fluid flow during metamorphism. Contrib. Mineral. Petrol..

[60] Yoshino T, Mibe K, Yasuda A, Fujii T (2002). Wetting properties of anorthite aggregates: Implications for fluid connectivity in continental lower crust. J. Geophys. Res. Solid Earth.

[61] Huang Y, Nakatani T, Nakamura M, McCammon C (2020). Experimental constraint on grain-scale fluid connectivity in subduction zones. Earth Planet. Sci. Lett..

[62] Kawano S, Yoshino T, Katayama I (2012). Electrical conductivity of magnetite-bearing serpentinite during shear deformation. Geophys. Res. Lett..

[63] Anders E, Owen T (1977). Mars and earth: Origin and abundance of volatiles. Science.

[64] McDonough WF (2003). Compositional model for the Earth’s core. Treatise Geochem..

[65] Smyth JR, Jacobsen SD, Jacobsen SD, Van Der Lee S (2006). Nominally anhydrous minerals and Earth’s deep water cycle. Earth’s Deep Water Cycle.

[66] Keppler H, Wiedenbeck M, Shcheka SS (2003). Carbon solubility in olivine and the mode of carbon storage in the Earth’s mantle. Nature.

[67] Shcheka SS, Wiedenbeck M, Frost DJ, Keppler H (2006). Carbon solubility in mantle minerals. Earth Planet. Sci. Lett..

[68] Luth R, Fei Y, Bertka CM, Mysen B (1999). Carbon and carbonates in the mantle. Mantle Petrology: Field Observations and High Pressure Experimentation (a Tribute to Francis R. (Joe) Boyd).

[69] Merlini M (2012). Structures of dolomite at ultrahigh pressure and their influence on the deep carbon cycle. Proc. Natl. Acad. Sci. U. S. A..

[70] Isshiki M (2004). Stability of magnesite and its high-pressure form in the lowermost mantle. Nature.

[71] Katsura T (1991). Stability of magnesite under the lower mantle conditions. Proc. Jpn Acad. Ser. B Phys. Biol. Sci..

[72] Gillet P (1993). Stability of magnesite (MgCO_3_) at mantle pressure and temperature conditions—A Raman-spectroscopic study. Am. Mineral..

[73] Redfern SAT, Wood BJ, Henderson CMB (1993). Static compressibility of magnesite to 20 GPa: Implications for MgCO_3_ in the lower mantle. Geophys. Res. Lett..

[74] Stagno V (2011). The stability of magnesite in the transition zone and the lower mantle as function of oxygen fugacity. Geophys. Res. Lett..

[75] Cannat M, Fontaine F, Escartín J, Rona PA, Devey CW, Dyment J, Murton BJ (2013). Serpentinization and associated hydrogen and methane fluxes at slow spreading ridges. Diversity of Hydrothermal Systems on Slow Spreading Ocean Ridges. Geophysical Monograph Series.

[76] Kong M, Lee Y (2019). Carbonation of chrysotile under subduction conditions. Engineering.

[77] Dódony I, Buseck PR (2004). Serpentines close-up and intimate: An HRTEM view. Int. Geol. Rev..

[78] Demichelis R, De La Pierre M, Mookherjee M, Zicovich-Wilson CM, Orlando R (2016). Serpentine polymorphism: A quantitative insight from first-principles calculations. CrystEngComm.

[79] Laporte D (1994). Wetting behavior of partial melts during crustal anatexis: The distribution of hydrous silicic melts in polycrystalline aggregates of quartz. Contrib. Mineral. Petrol..

[80] Laporte D, Rapaille C, Provost A, Bouchez JL, Hutton DHW, Stephens WE (1997). Wetting angles, equilibrium melt geometry, and the permeability threshold of partially molten crustal protoliths. Granite: From Segregation of Melt to Emplacement Fabrics.

[81] Manthilake G (2009). Electrical conductivity of wadsleyite as a function of temperature and water content. Phys. Earth Planet. Inter..

[82] Schiavi F (2018). Water quantification in silicate glasses by Raman spectroscopy: Correcting for the effects of confocality, density and ferric iron. Chem. Geol..

[83] Wang D, Karato SI (2013). Electrical conductivity of talc aggregates at 0.5 GPa: Influence of dehydration. Phys. Chem. Miner..

