# Supplementary material for: Insights on the deep carbon cycle from the electrical conductivity of carbon-bearing aqueous fluids
Source: Sci Rep. 2021 Feb 12;11:3745. doi: 10.1038/s41598-021-82174-8 (PMC7881151; doi:10.1038/s41598-021-82174-8)
Supplement: Supplementary file 1 — Supplementary Information. [file 41598_2021_82174_MOESM1_ESM.pdf]

Supplementary Information

Insights on the deep carbon cycle from the electrical conductivity of carbon-bearing aqueous fluids

Geeth Manthilake[1*], Mainak Mookherjee[2], Nobuyoshi Miyajima[3]

[1] Laboratoire Magmas et Volcans CNRS, IRD, OPGC, Université Clermont Auvergne, 63000 Clermont-Ferrand, France

[2] Earth Materials Laboratory, Department of Earth, Ocean and Atmospheric Sciences, Florida State University, Tallahassee, FL, 32306, USA

[3]Bayerisches Geoinstitut, University of Bayreuth, 95447 Bayreuth, Germany

[*]Corresponding author (geeth.manthilake@uca.fr)

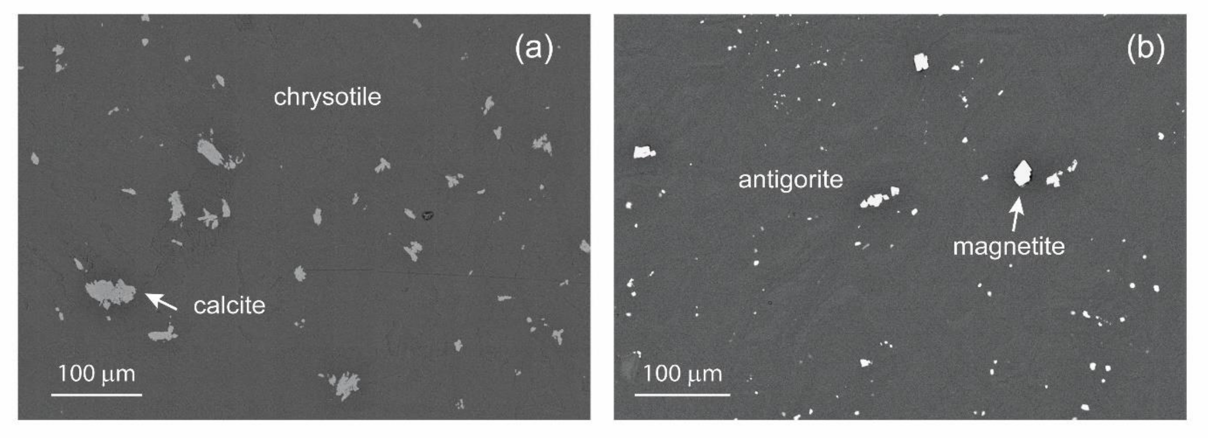

Supplementary Figure 1. The starting serpentine samples. The electron-backscattered images of the starting materials, (a) natural chrysotile, and the bright spots are calcite. (b) Natural antigorite with magnetite.

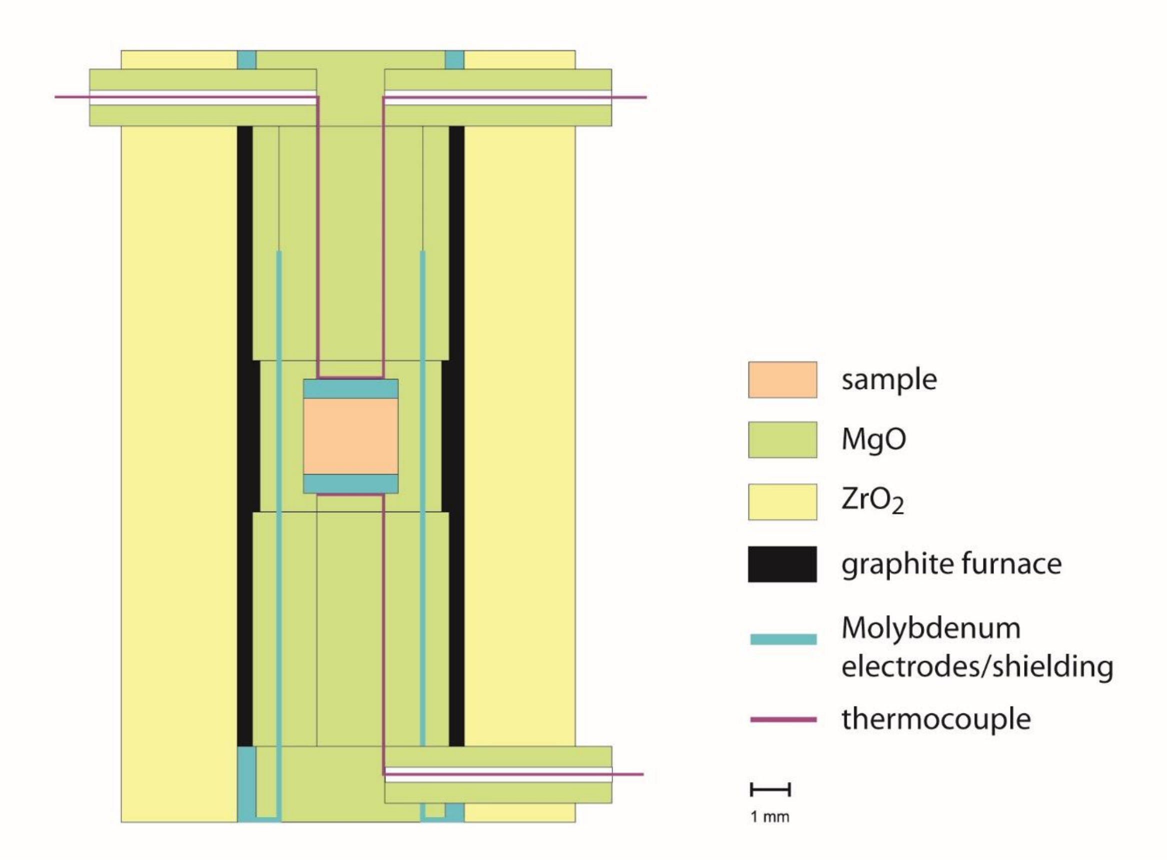

Supplementary Figure 2. The schematic cross-section of the multi-anvil cell-assembly for the in situ electrical conductivity measurements.

Supplementary Table S1. The chemical composition of starting serpentine samples

	Chrysotile	Calcite	Dolomite	Antigorite
**SiO_2**	42.49 (3)	0.46 (2)	0.07 (1)	41.35 (3)
**FeO**	1.59 (3)	0.09 (1)	0.32 (1)	6.28 (2)
**MgO**	40.84 (5)	0.49 (2)	21.88 (12)	36.54 (4)
**Al_2O_3**	0.14 (1)	n.d	n.d	1.78 (3)
**CaO**	n.d	55.12 (6)	35.92 (12)	n.d
**Total**	**85.08**	**56.17**	**58.20**	**85.96**

One (1σ) standard deviation uncertainty is reported in parenthesis as the last digit.
